# Supplementary material for: Diffusion Mediates Molecular Transport through the Perivascular Space in the Brain
Source: Int J Mol Sci. 2024 Feb 20;25(5):2480. doi: 10.3390/ijms25052480 (PMC10931628; doi:10.3390/ijms25052480)
Supplement: Supplementary file 1 [file ijms-25-02480-s001.zip › Legends to Supplementary Movie.pdf]

Legends to Supplementary Movie

Supplementary Movie S1. A continuous imaging of the penetrating artery at 80 μm deep for 20 minutes revealed gradual increase in FITC-dextran (40 kD dextran) concentration in the perivascular space (green). Most noticeably, the artery with its perivascular space changed its size and shape periodically. Intravascular lumen was visualized with SR-101 (red). These changes were also noted in Figure 2A.

Supplementary Movie S2. A time-lapse imaging of the penetrating vein at 20 μm deep for 10 minutes revealed gradual increase in FITC-dextran (40 kD dextran) concentration in the perivascular space (green). In contrast to the artery (Supplementary Movie 1), the vein as well as its perivascular space remained still without changes in its size and its shape. Intravascular lumen was visualized with SR-101 (red). These changes were also noted in Figure 2D.

Supplementary Movie S3. A reconstructed 3D image of penetrating artery and its branching arteriole from 20 to 50 μm deep at 30 minutes after superfusion of FITC-dextran (40 kD dextran) (green). Perivascular space was homogeneously visualized with dextran along vessels. Concentration gradient was not apparent in this depth difference. Intravascular lumen was visualized with SR-101 (red).
